# The interaction of the oxytocin receptor gene and child abuse subtypes on social cognition in euthymic patients with bipolar disorder type I

**DOI:** 10.3389/fpsyt.2023.1151397

**Published:** 2023-04-17

**Authors:** Ulises Ríos, Javier Morán, Jimena Hermosilla, René González, Paulina Muñoz, Marcelo Arancibia, Luisa Herrera, Juan Pablo Jiménez, Pablo R. Moya

**Affiliations:** ^1^Department of Psychiatry, School of Medicine, Faculty of Medicine, Universidad de Valparaíso, Valparaíso, Chile; ^2^Grupo de Investigación en Resiliencia, Adversidad Temprana y Reparación (GIRAR), Viña del Mar, Chile; ^3^Millennium Institute for Depression and Personality Research (MIDAP), Santiago, Chile; ^4^School of Psychology, Faculty of Social Sciences, Universidad de Valparaíso, Valparaíso, Chile; ^5^Mood Disorders Program, Hospital Psiquiátrico del Salvador, Valparaíso, Chile; ^6^Centro Interdisciplinario de Neurociencias de Valparaíso (CINV), Universidad de Valparaíso, Valparaíso, Chile; ^7^Mood Disorders Program, Hospital Dr Gustavo Fricke, Viña del Mar, Chile; ^8^School of Medicine, Faculty of Medicine, Universidad de Valparaíso, Valparaíso, Chile; ^9^Program of Human Genetics, Biomedical Sciences Institute, Universidad de Chile, Independencia, Chile; ^10^Department of Psychiatry, School of Medicine, Faculty of Medicine, Universidad de Chile, Independencia, Chile; ^11^Institute of Physiology, Faculty of Sciences, Universidad de Valparaíso, Valparaíso, Chile

**Keywords:** bipolar disorders, social cognition, child abuse, oxytocin receptors, environment

## Abstract

**Background:**

Most studies on cognitive impairment in bipolar disorder have neglected the role of early stress, despite the high frequency of childhood maltreatment in this clinical group. The aim of this study was to establish a connection between a history of emotional, physical, and sexual abuse in childhood and social cognition (SC) in patients with bipolar disorder type I (BD-I) in euthymia, and to test a possible moderating effect of the single nucleotide polymorphism *rs53576* in the oxytocin receptor gene (*OXTR*).

**Methods:**

One hundred and one participants were included in this study. History of child abuse was evaluated using the Childhood Trauma Questionnaire-Short Form. Cognitive functioning was appraised using The Awareness of Social Inference Test (social cognition). The interaction effect between the independent variables *OXTR rs53576* (AA/AG and GG) and the absence or presence of any one type of child maltreatment or a combination of types was analyzed using a generalized linear model regression.

**Results:**

BD-I patients who had been victims of physical and emotional abuse in childhood and were carriers of the GG genotype at *OXTR rs53576* displayed greater SC alterations, specifically in emotion recognition.

**Discussion:**

This gene–environment interaction finding suggests a differential susceptibility model of a genetic variants that can be plausibly associated with SC functioning and might help to identify at-risk clinical subgroups within a diagnostic category. Future research aimed at testing the interlevel impact of early stress constitutes an ethical-clinical duty given the high rates of childhood maltreatment reported in BD-I patients.

## 1. Introduction

Bipolar disorder (BD) has been linked to social cognition (SC) impairment, even during periods of euthymia (1, 2). Important heterogeneity exists, however, with measurable SC deficits being present in about one third of BD patients (3). Although most studies indicate that SC impairments do not predict functional outcomes beyond neurocognitive deficits in people with BD (4), there is also evidence suggesting that impairments in specific SC domains could be associated with psychosocial functioning in at least a subgroup of patients (5). Despite the vast literature on cognition impairments in BD, few authors have analyzed the impact of early stress (6). The lack of attention on this aspect may be relevant, given the importance of early stress on later neural development and cognitive functioning (7) as well as the substantial data demonstrating the high frequency of child abuse in BD patients (8, 9).

The few studies that have taken into account a history of childhood maltreatment in BD patients have reported significant differences in both SC and neurocognitive tests (10, 11). Greater cognitive deficits in BD patients with a history of childhood trauma may have a plausible biological basis (12).

### 1.1. Oxytocin, oxytocin receptor gene, and intermediate phenotypes

The oxytocin system regulates several processes involved in SC functioning (13), both in the cognitive route of SC—i.e., theory of mind (14) and emotion recognition (15, 16)- and in the affective route of SC—i.e., empathy (17). Some of these studies have examined genetic variations of the single nucleotide polymorphism *rs53576* (G/A), located in the third intron of the oxytocin receptor gene (*OXTR*), which has been linked to social behavioral phenotypes in humans (18).

There is a lack of work evaluating the hypothesis relating oxytocin to mood disorders in BD (19). Increased serum oxytocin levels in BD patients have been found in both symptomatic periods and euthymia (20). Given high phenotypic variability in BD, others have measured intermediate functioning dimensions, such as SC (21).

*OXTR* variants have been associated with outcomes at various levels, including schizophrenia (22), suicidal behavior (23) and depressive symptomatology (24). Meyer-Lindenberg et al. (25) evaluated associations of *OXTR* variants with intermediate phenotypes as a strategy to identify groups of patients with a common neurobiological substrate to target with more specific interventions. Studies that have taken this approach have reported an association between *OXTR* variants and SC functioning (26, 27).

Studies focused on intermediate phenotypes may be strengthened by designs that analyze gene–environment interactions. Most studies on *OXTR* variants have shown that the G allele of *rs53576* is associated with protective social traits, such as prosocial behavior (28) and greater empathic ability (17). However, authors who have evaluated its interaction with a background of early trauma have reported a pattern of greater vulnerability for this allelic variant. Studies on the interaction between *OXTR*, history of child abuse and emotional regulation in adult life have shown that carriers of the *rs53576* GG genotype display less emotional regulation if they had been victims of early trauma (29, 30). This pattern of differential vulnerability of the *OXTR rs53576* G allele has also been observed in functional imaging studies, which report strong gray matter reduction in the bilateral ventral striatum, along with increased amygdala responsiveness to emotional facial expressions (31). Malhi et al. (32) found that AA homozygotes *OXTR rs53576* carriers exposed to emotional trauma exhibited smaller left hippocampal volumes. Ebbert et al. (33) support the notion of being A carrier as a vulnerability factor. They reported, in a large cross-sectional study (*n* = 614), that the effect of emotional child abuse on family relationships during the adulthood was moderated by *OXTR rs53576*, observing that carriers of A allele had less supportive family relationships. However, not all studies support the relationship between child abuse, *OXTR rs53576* and depressive symptoms (34). Nevertheless, the available evidence on BD patients is extremely scarce.

Gene–environment interaction studies in patients with BD generally focus on neurocognitive measures and have involved other gene variants, such as BDNF val66met (35, 36). To the best of our knowledge, this is the first study to test a *OXTR-*SC gene–environment interaction in patients with BD. Considering the possible differential effects on brain development linked to subtypes of child abuse (37) and the evidence that certain subtypes of child abuse result in SC alterations (11, 38), we evaluated the association between a history of childhood abuse and SC functioning in patients with BD type I (BD-I) in euthymia, and the potential moderating effect of *OXTR rs53576*. We hypothesize that carriers of the G allelic variant will display a differential sensitivity pattern dependent on the subtype of child abuse.

## 2. Materials and methods

### 2.1. Sample

One hundred and one euthymic BD outpatients were recruited from the Mood Disorders Unit of Hospital Psiquiátrico del Salvador (Valparaíso, Chile) and the Outpatient Psychiatry Unit of Hospital Dr. Gustavo Fricke (Viña del Mar, Chile). Inclusion criteria were as follows: (a) BD-I DSM-IV-TR diagnosis, (b) age 18–65 years, (c) fulfilling euthymia criteria for at least 3 months, defined as a score of ≤6 on the Young Mania Rating Scale (YMRS) (39) and of ≤8 on the Hamilton Depression Rating Scale (HDRS) (40) and (d) the capacity to provide written informed consent. We excluded patients who were active drug users and who had received electroconvulsive therapy over the previous 3 months.

### 2.2. Procedure and instruments

The subjects’ history of child abuse was evaluated using the Spanish language version of the Childhood Trauma Questionnaire-Short Form (CTQ-SF) (41, 42). Briefly, this self-report instrument comprises 28 items that refer to five subtypes of child abuse: sexual abuse (SA), emotional abuse (EA), physical abuse (PA), physical neglect, and emotional neglect. For ethical-clinical reasons, the self-report instrument was completed by each patient in a clinical setting, confidentially and in the presence of their treating physician. We only considered the abuse scales, as recommended by Aas et al. (36).

Cognitive functioning was evaluated with social and non-social cognition tests. For the former dimension, we used the Spanish language version of The Awareness of Social Inference Test (TASIT) (43), specifically the emotion evaluation task. This test evaluates the ability to recognize emotions by showing 20 micro-videos in which actors simulate situations that represent the basic emotions of fear, disgust, surprise, sadness, and anger. Neurocognitive aspects were evaluated with the Addenbrooke’s Cognitive Examination-Revised (ACE-R), Chilean version (44).

This study was approved by the research ethics committee of the Valparaíso-San Antonio Health Service, Chile. All patients were informed about the study and gave their signed consent.

### 2.3. Genotyping

After the interview, blood samples (6 ml) were taken from the patients and placed in EDTA-coated tubes. The DNA extraction was performed using the NucleoSpin Blood kit (Macherey-Nagel, Germany) following manufacturer’s protocol. The allelic *OXTR rs53576* variants were genotyped using a TaqMan® probe (SNP ID C___3290335_20, Applied Biosystems, USA) in a AriaMX thermocycler (Agilent, USA) following the manufacturer’s protocol. Hardy–Weinberg equilibrium was tested by comparing the observed and expected genotypes using *χ*^2^.

### 2.4. Statistical analysis

Using generalized linear model regression, we evaluated the interaction effect between the independent variables *OXTR rs53576*, coded as AA/AG (0) and GG (1), and the absence (0) or presence (1) of any one type of child abuse or a combination of types. We used cut-off scores for “moderate” to “extreme” levels in the CTQ for SA (higher than 7 points), PA (higher than 9 points), and EA (higher than 12 points). The dependent variable was the total score on the TASIT.

First, we sought to identify if the presence of any subtype of abuse had an interaction effect with *OXTR* on SC. Second, to evaluate whether specific differences associated with each subtype of abuse, we repeated the analysis to evaluate the interaction between *OXTR* and the CTQ-SF subscales. In addition, as a way of assessing the presence of combinations of abuse subtypes, we generated three new variables: PA + SA; PA + EA; SA + EA. Lastly, we conducted a factorial ANOVA, for which we generated an ordinal variable based on abuse subtypes and combinations found to have interaction effects in the previous step.

## 3. Results

### 3.1. Frequency of subtypes of child abuse and allelic frequency of *OXTR rs53576*

Participants were 45.5 years old on average (13.9 SD); 65.7% of them were women. 64% of the patients reported being victims of at least one type of abuse as a child. The frequencies *per* subtype of abuse were 42% for SA, 30% for PA and 23% for EA (Table 1). Regarding abuse combinations, 10% of the patients were victims of three subtypes of abuse and 17% of at least two subtypes. Our analysis by sex showed that 71% of the women and 51% of the men had suffered at least one type of abuse as children (*p* < 0.02).

**Table 1:** Demographic and clinical characteristics of study sample.

	OXTR GG *n* = 20 (19.8%)	OXTR GA *n* = 71/AA *n* = 9 (80.2%)	Value of *p*
Sex (woman)	12 (60.0%)	49 (60.5%)	*χ*^2^ = 0.904
Age (years)	47.4 (12.0)	47.2 (15.7)	*t* = 0.949
ACE-R	87.0 (1.0)	85.2 (9.4)	*t* = 0.459
Onset (years)	23.3 (8.2)	23.2 (10.2)	*t* = 0.960
Number of hospitalizations	3.7 (4.5)	3.6 (4.5)	*t* = 0.959
Sexual abuse (1)†	9	22	*χ*^2^ = 0.057
Emotional abuse (1)†	7	21	*χ*^2^ = 0.273
Physical abuse (1)†	3	19	*χ*^2^ = 0.505

†, presence of abuse; ACE-R, Addenbrooke’s Cognitive Examination-Revised.

We genotyped the *rs53576* single nucleotide polymorphism located in *OXTR*. This variant was not in Hardy–Weinberg equilibrium (*χ*^2^ = 19.8, value of *p* = 0.000009), which might be explained by sample selection: patients with BD-I and not the general population. This is plausible as *rs53576* has been linked to several behavioral traits and may contribute to the phenotype under study. The genotype frequencies of *OXTR rs53576* were 20% GG and 80% GA + AA; we did not find differences by sex or age. There were no differences in the age of BD onset or number of hospitalizations.

### 3.2. Gene–environment interaction and SC functioning by presence of abuse (step 1)

Presence of abuse, determined by subscales of the CTQ-SF that surpass the score for moderate abuse, was not associated either as a simple effect (*β* = −0.565; *p* = 0.465) and did not show interaction with *OXTR* allelic variants (*β* = 1.802; *p* = 2.45; Table 2).

**Table 2:** Gene–environment interaction and SC functioning by presence of abuse.

Step	Model	Variable	*β*	95% CI		*p*
Step 1						
		Intercept	15.696	14.943	1.645	< 0.001
		GG/AGAA	0.094	−1.430	0.162	0.904
		Abuse (1)†	−0.565	−2.071	0.942	0.465
		GG/AGAA ✻ Abuse	1.802	−1.209	0.481	0.245
Step 2						
	1 Abuse model	(Intercept)	15.650	1.503	16.280	< 0.001
		GG/AGAA	0.479	−0.770	1.730	0.455
		EA	−0.089	−1.338	1.160	0.888
		GG/AGAA ✻ EA	2.614	0.117	5.110	0.044
						
		(Intercept)	1.485	13.994	1.571	< 0.001
		GG/AGAA	0.206	0.321	0.380	0.023
		PA	−0.154	−3.239	0.165	0.081
		GG/AGAA ✻ PA	0.438	0.982	0.778	0.014
						
		(Intercept)	1.545	14.799	1.609	< 0.001
		GG/AGAA	0.493	−0.819	0.181	0.464
		SA	0.191	−1.095	0.148	0.772
		GG/AGAA ✻ SA	−0.814	−3.477	0.185	0.551
						
	2 Abuses model	(Intercept)	15.766	14.841	1.669	< 0.001
		GG/AGAA	0.440	−1.431	−1.431	0.231
		EA + SA	0.823	−1.024	0.267	0.386
		GG/AGAA ✻ EA + SA	−0.053	−3.780	0.368	0.978
						
		(Intercept)	1.541	14.186	1.664	< 0.001
		GG/AGAA	0.748	−1.742	0.324	0.558
		PA + SA	−0.128	−2.562	0.231	0.918
		GG/AGAA ✻ PA + SA	0.550	−4.369	0.547	0.827
						
		(Intercept)	1.490	14.021	1.579	< 0.001
		GG/AGAA	0.213	0.363	0.391	0.021
		EA + PA	−0.149	−3.246	0.265	0.101
		GG/AGAA ✻ EA + PA	0.445	0.963	0.794	0.015
Step 3						
	Ordinal model	(Intercept)	15.329	14.534	1.613	< 0.001
		GG/AGAA	1.440	−0.165	0.305	0.085
		1 EA or PA	0.094	−1.645	0.183	0.916
		2 EA & PA	−1.866	−3.913	0.181	0.080
		AGAA/GG ✻ 1 EA or PA	1.424	−2.044	0.489	0.425
		AGAA/GG ✻ 2 EA & PA	5.169	1.090	0.925	0.016

†, presence of abuse; EA, emotional abuse; PA, physical abuse; SA, sexual abuse.

### 3.3. Gene–environment interaction and SC functioning by specific types of abuse (step 2)

The lacck of a simple interaction effect between the variables under study prompted the evaluation of the type of child abuse and its interaction with *rs53576* genotypes. None of the subtypes of abuse displayed a simple association effect with SC. EA (*β* = 2.614; *p* = 0.044) and PA (*β* = 0.438; *p* = 0.014) displayed an interaction effect with the genetic variable. When evaluating whether the sum of two types of abuse interacted with *OXTR*, only PA + EA displayed an association with SC (*β* = 0.445; *p* = 0.015).

### 3.4. Gene–environment interaction and SC functioning by specific types of abuse (step 3)

**Figure 1:**
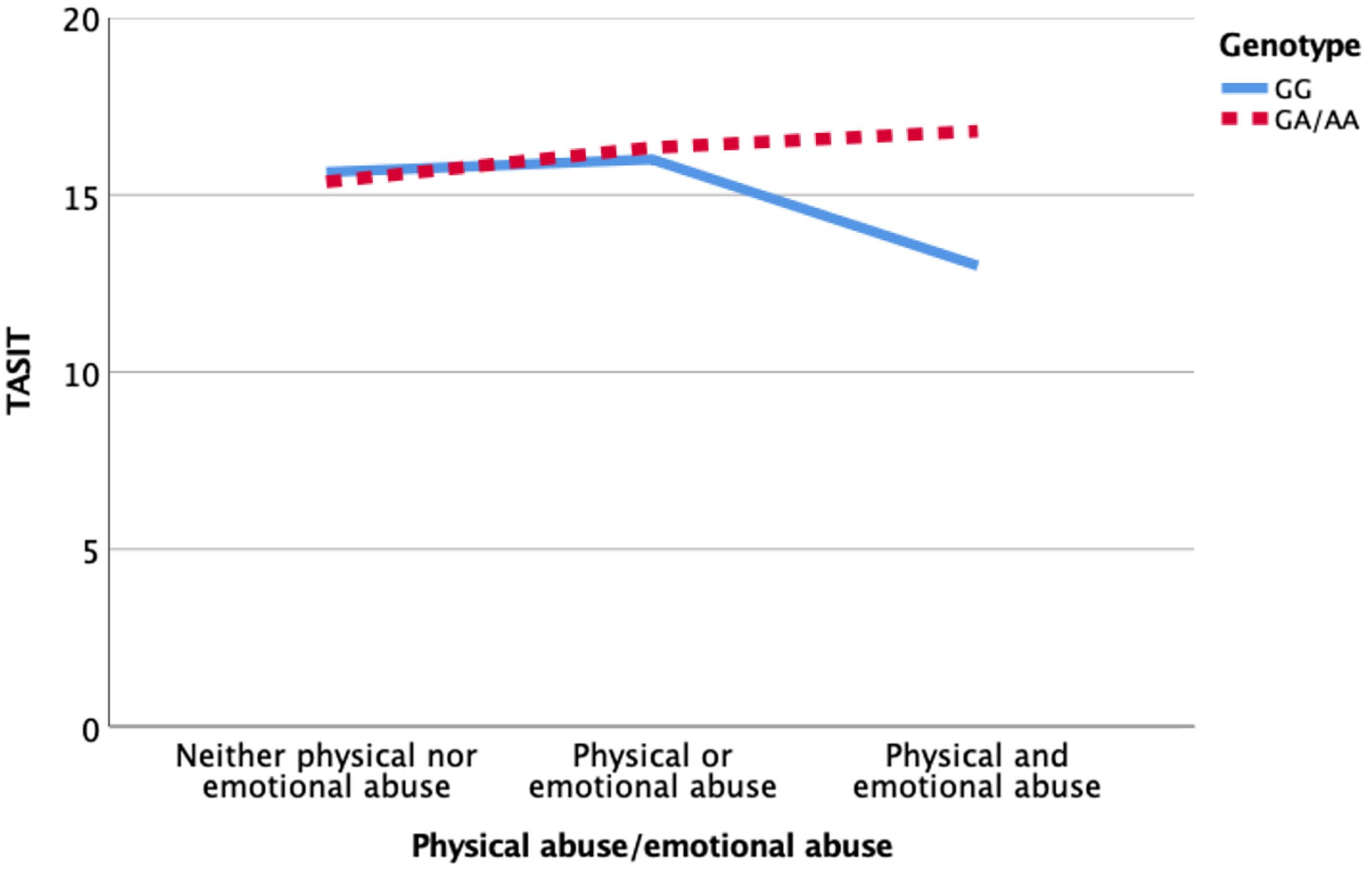
Impact of the interaction between *OXTR rs53576* (GG vs. GA/AA), physical abuse, and emotional abuse on performance on an emotion recognition test (TASIT).

An ordinal variable was generated: we coded EA and PA as “1 abuse type” and EA + PA as “2 abuse types.” Although the presence of 1 abuse type is not associated with differences between the different *rs53576* genotypes (*β* = 1.424; *p* = 4.25), the presence of 2 abuse types demonstrated altered emotion recognition scores in BD-I patients who carry the GG genotype (*β* = 5.169; *p* = 0.016; Figure 1).

## 4. Discussion

Our study revealed that euthymic patients with BD-I that were victims of both PA and EA as children and that were carriers of the *OXTR rs53576* GG genotype displayed greater SC alterations, especially in an emotion recognition test.

In our sample, the frequencies of different types of abuse were remarkably high: 42% for SA, 30% for PA and 23% for EA. These frequencies are similar to those found by Vitriol et al. (45) in a prevalence study with a Chilean sample of patients with depressive disorder (*n* = 440). They found that 82% had experienced at least one traumatic childhood event, i.e., 49% domestic violence and 40% sexual abuse. In a systematic review by Maniglio et al. (46) including 3,407 BD patients (from 20 studies), a prevalence of 24% was found for SA. The authors state that this rate might be underestimated, since many studies restricted their definitions to the most severe forms of SA. In addition, the findings suggest that BD patients exhibited higher rates of SA in comparison to healthy individuals. Post et al. (47) showed an incidence of 50% of child abuse in BD patients (22% of SA in the entire sample; up to 36% in the US).

Our findings support the hypotheses of previous studies (29, 30) suggesting that the *OXTR rs53576* GG genotype constitutes a differential susceptibility genotype (48) when dealing with early stress conditions. The gene–environment interaction model has been confirmed in studies focused on outcomes belonging to various levels including increased depressive symptomatology (49), alterations in emotional regulation tests (29, 30), and morpho-functional modifications in the limbic system (31).

The present study adds evidence for gene–environment interaction, focusing on an intermediate phenotype such as SC functioning, and thus providing data that may improve our comprehension of previous hypotheses centered on more complex variables. In other words, alterations in emotional recognition tasks may well be related to greater demands on emotional regulation processes and their morpho-functional correlates in the limbic system. Such a clinical context may also shed light on why depressive symptomatology indexes are greater in carriers of the *OXTR rs53576* GG genotype who have been affected by child abuse.

Our findings also highlight differences on the role of the child abuse subtypes. Most studies have employed general measures of child abuse, neglecting the more specific characteristics of the traumatic experience and its possible differential effects on the brain (37). Our results revealed an interaction only in the presence of PA and EA, with no interaction with SA, despite the evidence indicating its undeniable impact on the life cycle of BD patients (46). These results are consistent with previous studies that have reported an association between the PA and EA subtypes and SC performance, specifically in emotional recognition tests (11, 38). In light of these results, the hypothesis that SA experiences have a course in a person’s development that differs from that of other types of abuse (50) should be taken into account in future research. Nevertheless, due to limitations such as our relatively small sample size and methodological aspects of the genotype grouping (GG vs. GA + AA), these hypotheses should be weighed with caution. Similarly, interpretation of the results should be cautious given the limited information on the more specific characteristics of each person’s traumatic experience during childhood. As suggested in studies on the effect of early interventions (51), this variable may attenuate or revert the long-term impact on brain function. Similarly, future research should consider the age at which the abuse occurred, as there is evidence of differential vulnerability in specific brain regions depending on the period of neural development period in which the stressor occurs (52).

The relevance of generating evidence, specifically in relation to BD, lies in the fact that cognitive alterations in this group have led to the design of therapeutic interventions and preventive strategies (53) that have overlooked the role of early stress. Most recommended therapeutic interventions are based on the assumption that this diagnostic category carries an inherent cognitive deficit, presuming that neuroprogression occurs as a consequence of the allostatic load of the disease (54). This perspective might reflect a possible biomedical bias, which has omitted the analysis of early psychosocial variables in studies of cognitive dysfunction in BD.

Among the limitations of our study, the assessment of traumatic episodes such as child abuse could be limited by characteristics of the instrument used that do not allow a sufficiently precise and deep characterization among the subtypes of maltreatment. In addition, TASIT was the only measure of SC and ACE-R might be a limited test when examining neurocognitive functioning in BD patients. On the other hand, a larger sample size might have allowed a more adequate analysis of the association between other dimensions of SC and *OXTR* variants.

Our findings highlight the need to rethink the design of new therapeutic approaches from a preventive perspective, with strategies aimed at reducing the incidence of child abuse in populations known to be at higher risk (8, 9) as well as through interventions aimed at fostering specific emotional regulation and mentalization skills (55) from a relational perspective. Conducting research aimed at testing interventions that reduce the inter-level impact of early stress (56) represents an ethical-clinical imperative in light of the high rates of child abuse in patients with BD.

This study has pioneered the exploration of SC in patients with BD-I using a gene–environment interaction model. Our aim was to provide data about SC deficits employing a hypothesis that presumes that adverse relational events in early life have an impact and interact with genetic variants of a plausibly related polymorphism such as *OXTR rs53576*. The description of a differential susceptibility genotype associated with the functioning of intermediate phenotypes may contribute to the identification of at-risk clinical subgroups within a diagnostic category with well-established intra-group heterogeneity such as BD.

## Data availability statement

The raw data supporting the conclusions of this article will be made available by the authors, without undue reservation.

## Ethics statement

The studies involving human participants were reviewed and approved by Comite etico cientifico del Servicio de Salud Valparaiso San Antonio Chile. The patients/participants provided their written informed consent to participate in this study.

## Author contributions

UR designed the study and managed the literature searches. JH and PM were in charge of administering the questionnaires. PRM and RG were in charge of genetics analysis. JM was in charge of the statistical analysis. All authors contributed to the article and approved the submitted version.

## Funding

The study receive funding from ANID - Millennium Science Initiative Program/Millennium Institute for Research on Depression and Personality-MIDAP ICS13_005.

## Conflict of interest

The authors declare that the research was conducted in the absence of any commercial or financial relationships that could be construed as a potential conflict of interest.

## Publisher’s note

All claims expressed in this article are solely those of the authors and do not necessarily represent those of their affiliated organizations, or those of the publisher, the editors and the reviewers. Any product that may be evaluated in this article, or claim that may be made by its manufacturer, is not guaranteed or endorsed by the publisher.
